# Association between the *BRCA2 *N372H variant and male breast cancer risk: a population-based case-control study in Tuscany, Central Italy

**DOI:** 10.1186/1471-2407-7-170

**Published:** 2007-09-03

**Authors:** Domenico Palli, Mario Falchetti, Giovanna Masala, Ramona Lupi, Francesco Sera, Calogero Saieva, Cristina D'Amico, Marco Ceroti, Piera Rizzolo, Maria Adelaide Caligo, Ines Zanna, Laura Ottini

**Affiliations:** 1 Molecular and Nutritional Epidemiology Unit, CSPO, Scientific Institute of Tuscany, 50139 Florence, Italy; 2 Department of Experimental Medicine, University of Rome "La Sapienza", 00161 Rome, Italy; 3 Division of Surgical, Molecular and Ultrastructural Pathology, Department of Oncology, University of Pisa and Pisa University Hospital, 56126 Pisa, Italy

## Abstract

**Background:**

Male breast cancer (MBC) is a rare disease and little is known about its aetiology. Germ-line mutations of *BRCA2 *and, at lower frequency, of *BRCA1 *are implicated in a relatively small proportion of MBC cases. Common polymorphic variants in *BRCA1 *and *BRCA2 *genes may represent breast cancer (BC) susceptibility alleles and could be associated with a modestly increased risk of MBC at population level. Considering the relevant role of *BRCA2 *in MBC, we investigated whether the *BRCA2 *N372H variant, representing the only common non-synonymous polymorphism in *BRCA2*, might modulate the risk of BC in male populations.

**Methods:**

A case-control study was performed comparing a population-based series of 99 MBC cases, characterized for *BRCA1 *and *BRCA2 *mutations, with 261 male population controls, all residing in Tuscany, Central Italy. All MBC cases and controls were genotyped for the *BRCA2 *N372H allele by TaqMan allelic discrimination assays. To evaluate the genotype specific risk of the *BRCA2 *N372H variant, MBC carriers of germ-line *BRCA1/2 *mutations were excluded from the analyses.

**Results:**

No association emerged in univariate and age-adjusted analyses. Age-specific analyses suggested an increased risk for the HH homozygous genotype in subjects younger than 60 years. A statistically significant interaction emerged between this genotype and age (p = 0.032). When analyses were restricted to MBC cases enrolled in the first 4 years following diagnosis, a recessive model showed a significantly increased risk of MBC in HH subjects younger than 60 years (OR = 5.63; 95% CI = 1.70;18.61).

**Conclusion:**

Overall, our findings, although based on a relatively small series, suggest that the *BRCA2 *HH homozygous genotype might be positively associated with an increased risk of MBC in men younger than 60 years.

## Background

Male breast cancer (MBC) is a rare disease and little is known about its aetiology compared with female breast cancer (FBC). MBC represents less than 1% of all cancers in men and its incidence is increasing in younger men [1]. In Italy, MBC accounts for 0.2% of all cancers in males [2].

Similar to FBC, a positive family history (FH) of breast cancer (BC) is associated with increased risk of MBC and approximately 15% to 20% of male patients with BC have a positive FH [3]. The two major hereditary BC genes, *BRCA1 *(OMIM #113705) and, to a larger extent, *BRCA2 *(OMIM # 600185), are implicated in MBC. Both genes are estimated to be responsible respectively for up to 16% and 76% of the MBCs in high-risk breast/ovarian cancer families [3,4]. The frequencies of *BRCA1/BRCA2 *mutations are sharply different in ethnically diverse population- and clinic-based series ranging from 0% to 4% for *BRCA1 *and from 4% to 40% for *BRCA2 *[5,6]. However, at population level only a small proportion of all MBC cases are due to inherited mutations in *BRCA1/BRCA2 *genes.

Low-penetrance polymorphisms in BC susceptibility genes are present in a high percentage of individuals and might account for BC risk at population level. In this respect, common polymorphic variants in *BRCA1/BRCA2 *genes may represent BC susceptibility alleles and could be associated with a modest risk of MBC that would explain, on population basis, a large proportion of the disease.

The N372H (rs144848) polymorphism is the unique variant in *BRCA2 *gene that results in an amino acid change and has a rare allele frequency greater than 10%. Little is known on the functional effect of this polymorphism, although the substitution of asparagine (a neutral amino acid) by histidine (a basic amino acid) may be expected to affect BRCA2 structure and function as it falls in a region that has been shown to interact with the histone acetyltransferase P/CAF prior to transcriptional activation of other genes [7]. Intriguingly, an excess of NH heterozygotes was observed in female controls and an effect of this variant on fetal survival in a sex-dependent manner has been suggested as newborn females showed an excess of heterozygotes and a deficit of homozygotes, whereas the opposite was observed in newborn males [8].

Several studies have analysed the effect of the *BRCA2 *N372Hpolymorphism on the risk of breast cancer in female populations with inconsistent findings. The HH homozygous genotype was reported to be associated with a 1.3- to 1.5-fold increased risk of breast and ovarian cancer [8-11]. In contrast, other studies did not show any effect of this polymorphism on breast or ovarian cancer risk [12-15]. A recent large pooled analysis, however, could not exclude an effect of this genotype in younger women [16]. To our knowledge, the effect of the *BRCA2 *N372H polymorphism on BC risk has not yet been investigated in male populations.

Considering the relevance of *BRCA2 *in MBC, we have performed a population-based case-control study to investigate the role of *BRCA2 *N372H variant on BC risk in men, comparing a series of MBC, characterized for *BRCA1 *and *BRCA2 *mutation status, with a control group from the same area of Central Italy (Florence, Tuscany).

## Methods

### Study population and data collection

In a previous study [17], we had identified a population-based series of 25 MBC cases diagnosed in the area of Florence (Tuscany, Central Italy) during the period 1990–1998. One of the problems in MBC studies is represented by the rarity of the disease: in order to increase the number of cases for meaningful statistical analyses it is necessary to enrol patients diagnosed over a long period of time (or in a larger geographical area). Thus, using all available local sources (including Pathology Departments and the Hospital Discharge database) the original series has been expanded and 74 additional MBC cases diagnosed in the period 1991–2006 have been enrolled for a total of 99 MBCs available for this study, all residing in Tuscany.

Overall, after exclusion of deceased and migrated patients, 85 additional unrelated MBC were traced and invited to participate into the study. Eleven cases refused to participate, mostly because of advanced age or severe illness. Confirming our previous response rate of about 80%, 74 of these 85 MBC cases (87.1%) agreed to participate. According to the original study protocol, each MBC patient provided: 1) informed consent; 2) blood samples; 3) detailed information on his personal and family history of cancer at any site, including all first- and second-degree relatives of both genders; 4) a detailed smoking and working history up to the date of BC diagnosis. Information has been validated by available sources (mainly local Cancer and Mortality Registries). Procedures to maintain confidentiality for all the information collected have been developed and strictly applied. The study was approved by the local Ethical Research Committee (Florence Health Unit).

### Population controls

In the frame of a multi-site epidemiological project, two series of healthy adults of both sexes have been randomly selected from the municipality lists of two areas in Tuscany: the city of Florence and one rural area [18,19]. All participants signed an informed consent form and provided a blood sample. Out of 700 randomly selected subjects, 553 subjects (79%) accepted to participate into the study; males were 48.6% (269/553) and 261 samples were available for analyses (eight of the initial 269 control's samples (3%) yielded insufficient DNA). The blood samples have been processed at the study laboratory (CSPO, Florence), in the same day of collection and divided in specific aliquots (RBC, buffy coat, serum, plasma). The aliquots have been stored in a -80°C freezer at the Biological Bank of the Molecular Epidemiology Unit at CSPO.

### Mutational analysis

Buffy coat aliquots from MBC cases and controls were anonymously shipped to the research laboratory (Experimental Medicine, Rome) where genomic DNA was extracted using QIAamp DNA Mini Kit (Qiagen Inc., Charlesworth, CA). The entire *BRCA1 *and *BRCA2 *coding sequences, including intron-exon boundaries, were analysed by using single strand conformation polymorphisms (SSCP) combined with protein truncation test (PTT) and automatic sequencing analysis [17,20]. Mutations were always verified by PCR direct sequencing on two independent blood samples (reference sequence for *BRCA1*: Genbank, U14680; reference sequence for *BRCA2*: Genbank, NM_000059).

### SNP genotyping

The *BRCA2 *N372H (rs144848) polymorphism was analysed by TaqMan allelic discrimination using Real-Time PCR. Fluorescent hybridisation probes, labelled with 6-carboxyfluorescin (FAM) to detect the 1342C (372H) allele and with Texas Red to detect the 1342A (372N) allele were specifically designed. TaqMan assays were performed in a reaction volume of 50 μl, comprising 100 ng of DNA, 400 nM each probe, 400 nM each primer and 1 × Universal Master Mix (Biorad). The following conditions were used for amplification: 3 minutes at 95°C and 55 cycles at 95°C for 10" and 63°C for 45". The Real-Time PCR was accomplished on iCycler thermal cycler (Biorad) with 96 × 0.2 ml samples. The fluorescence was visualised through iCycler Optical System (Biorad) during Real-Time PCR and analysed by using the iCycler IQ Real-Time Detection System (Biorad) 3.0 software. About 15% of the genotyping results were confirmed by sequencing analysis. The genotype controls for the three possible genotypes plus "no template" controls were always included in each analysis.

### Statistical analysis

Allele frequencies have been calculated as the number of alleles divided by the number of chromosomes. Genotype frequencies have been calculated as the number of participants with a particular genotype divided by the number of participants. Tests for Hardy-Weinberg equilibrium among cases and controls have been assessed using Pearson's χ^2 ^test with one degree of freedom comparing expected genotype frequencies (based on observed qs) to observed genotype frequencies. The association between MBC risk and *BRCA2 *N372H was measured by the odds ratio (OR) and its corresponding 95% confidence interval and was estimated using unconditional logistic regression after adjustment for age considered as dichotomous variable (<60 years, >60 years). The analysis were performed with four logistic regression models based on a co-dominant, dominant, recessive and multiplicative codominant effect (inheritance model). In the dominant model, both the heterozygous variant and rare homozygous variant were combined in a dummy variable. In the recessive model, the variant was defined in a dummy variable as only the rare homozygous genotype. In the co-dominant model both rare homozygous and heterozygous variant effects were estimated using two dummy variables while in the multiplicative codominant model a dose-response effect were tested on the variable counting the number of copies of the H allele. Analyses have also been carried out according to stratification by age at diagnosis for cases and age at interview for controls (below/above 60 years) and restricted to MBC cases with a time interval after diagnosis shorter than the median value of the series (4 years). Interactions between age, modelled as dichotomous variable (<60 years, >60 years), and genotype under co-dominant, dominant, recessive and multiplicative codominant models, were assessed using Likelihood Ratio test (LR) comparing logistic regression models with and without interaction term.

## Results

In order to evaluate the putative influence of the *BRCA2 *N372H polymorphism on MBC risk, we carried out a population-based case-control study based on a total of 99 MBC cases and 261 adult male controls from the same area of Central Italy (Florence, Tuscany). The mean age at interview was 67.9 (SD 11.8) in cases and 55.9 (SD 6.9) in controls (p < 0.0001). The mean age at diagnosis in the MBC series was 63.4 (SD 12.1; median 65) and the time interval between diagnosis and interview ranged from 0 to 26 years (median 4.0). A detailed FH for breast and ovarian cancers diagnosed in first-degree relatives was collected for all MBC cases. Overall, 25.3% (25/99) of MBC patients reported a first degree FH positive for breast/ovarian cancer.

MBC cases were characterized for *BRCA1 *and *BRCA2 *mutations. Overall, 8/99 (8.1%) MBC cases resulted to be mutated in *BRCA1/BRCA2 *genes: 6 cases (6.1%) carried *BRCA2 *mutations and 2 cases (2.0%) carried *BRCA1 *mutations. All 99 MBC cases and 261 controls were analysed for the *BRCA2 *N372H allele and genotype frequencies. Genotype distribution was consistent with Hardy-Weinberg equilibrium (p = 0.529) among our population controls and cases (p = 0.067). In order to evaluate the genotype specific risk of the *BRCA2 *N372H polymorphism, MBC carriers of germ-line *BRCA1/2 *mutations (8 cases) were excluded from the analyses.

As shown in Table 1, no statistically significant difference in the distribution of the three specific *BRCA2 *N372H genotypes was observed between MBC cases and controls (p = 0.46). The age-adjusted analysis of the genotype-specific risks showed that individuals with NH heterozygous and HH homozygous genotypes were not at increased MBC risk. These results were confirmed on dominant, recessive and multiplicative codominant transmission models (data not shown). Separate analyses stratified according to age (below/above 60 years) showed a tendency towards an inverse association between the H allele and MBC risk in the older sub-group (age > 60 years), although far from being statistically significant. On the contrary, an increased MBC risk (OR = 3.12; 95%CI = 1.08; 9.03) was observed in younger subjects (age ≤ 60 years) on the basis of a recessive model (Table 2). A model specifically aimed to evaluate a possible effect modification of age on the association between MBC risk and the HH homozygous genotype found a statistically significant interaction (LR test p = 0.02; OR_interaction _= 0.17; 95%CI = 0.04; 0.76).

**Table 1:** Distribution of 91 MBC cases not mutated in BRCA genes and 261 male population controls according to the *BRCA2 *N372H genotype frequencies.

Genotype	Cases n	%	Controls n	%	Co-dominant model OR 95% CI
N/N	48	52.7	127	48.7	
N/H	31	34.1	107	41.0	0.68 (0.40; 1.18)
H/H	12	13.2	27	10.3	0.94 (0.43; 2.09)
Total	91	100.0	261	100.0	

Odds Ratios (OR) and 95% Confidence Intervals according to a co-dominant model, estimated by a logistic regression model after adjustment for age considered as a dichotomous variable (<60 years, >60 years).

**Table 2:** Distribution of 91 MBC cases not mutated in BRCA genes and 261 male population controls according to the *BRCA2 *N372H genotype frequencies, stratified by age at diagnosis.

Genotype	Cases n	%	Controls n	%	Co-dominant model OR 95% CI	Recessive model OR 95% CI
*≤60 years*						
N/N	16	50.0	94	54.0		
N/H	10	31.2	68	39.1	0.86 (0.37; 2.03)	
H/H	6	18.8	12	6.9	2.94 (0.94; 9.15)	3.12 (1.06; 9.16)
Total	32	100.0	174	100.0		
*>60 years*						
N/N	32	54.2	33	37.9		
N/H	21	35.6	39	44.8	0.56 (0.27; 1.15)	
H/H	6	10.2	15	17.2	0.41 (0.14; 1.22)	0.54 (0.20; 1.51)
Total	59	100.0	87	100.0		

Odds Ratios (OR) and 95% Confidence Intervals according to co-dominant and recessive models, estimated using logistic regression models. Interaction among between BRCA2 N372H genotype and age: OR_interaction _= 0.17 (95%CI = 0.04–0.76); p = 0.02.

We carried out additional analyses taking into account the length of the interval between diagnosis and blood donation among cases. Overall, no association between the H allele and MBC risk was observed, when analyses were restricted to a comparison between the group of 53 MBC cases with an interval shorter than the median value of the whole series (≤4 years) and the series of controls (Table 3). Additional analyses stratified by age, showed a strongly increased MBC risk (OR = 5.63; 95%CI = 1.70; 18.61) in younger subjects with the HH homozygous genotype (Table 4), while no statistically significant result emerged in older individuals. Further statistical analyses confirmed that the effect of the HH homozygous genotype on MBC risk tended to be modified by age also in these analyses restricted to MBC cases with a shorter time interval between diagnosis and blood donation (LR test p = 0.03; OR_interaction _= 0.17 (95%CI = 0.04–0.83). Sensitivity analyses were carried out based on cut-off points for both age at diagnosis and time interval after diagnosis different from those identified *a priori*. These analyses confirmed the association, when older individuals (up to 65 years of age) or cases with a longer time interval (up to 6 years) were included.

**Table 3:** Distribution of 53 MBC cases with a time interval between diagnosis and interview ≤4 years and 261 male population controls according to the *BRCA2 *N372H genotype frequencies.

Genotype	Cases n	%	Controls n	%	Co-dominant model OR 95% CI
N/N	24	45.2	127	48.7	
N/H	18	34.0	107	41.0	0.79 (0.40; 1.58)
H/H	11	20.8	27	10.3	1.67 (0.70; 3.97)
Total	53	100.0	261	100.0	

Odds Ratios (OR) and 95% Confidence Intervals according to a co-dominant model, estimated using logistic regression models after adjustment for age considered as a dichotomous variable (<60 years, >60 years).

**Table 4:** Distribution of 53 MBC with a time interval between diagnosis and interview ≤4 years and 261 male population controls according to the *BRCA2 *N372H genotype frequencies, stratified by age at diagnosis.

Genotype	Cases n	%	Controls n	%	Co-dominant model OR 95% CI	Recessive model OR 95% CI
*≤60 years*						
N/N	6	35.3	94	54.0		
N/H	6	35.3	68	39.1	1.38 (0.43; 4.47)	
H/H	5	29.4	12	6.9	6.53 (1.73; 24.69)	5.63 (1.70;18.61)
Total	17	100.0	174	100.0		
*>60 years*						
N/N	18	50.0	33	37.9		
N/H	12	33.3	39	44.8	0.56 (0.24;1.34)	
H/H	6	16.7	15	17.2	0.73 (0.24;2.22)	0.96 (0.34;2.71)
Total	36	100.0	87	100.0		

Odds Ratios (OR) and 95% Confidence Intervals according to co-dominant and recessive models estimated using logistic regression models. Interaction between BRCA2 N372H genotype and age: OR_interaction _= 0.17 (95%CI = 0.04–0.83); p = 0.03.

## Discussion

We evaluated the effect of the *BRCA2 *N372H (1342A > C) polymorphism on MBC risk in a population based case-control study. Overall, a positive association between the HH genotype and an increased risk of MBC was suggested in men younger than 60 years; this association was particularly strong (with a five-fold increased risk) when analyses were restricted to 53 MBC cases enrolled in the study with a blood donation not later than 4 years after their diagnosis. To our knowledge, the present study is the first reporting an analysis of the association between BC risk and the *BRCA2 *N372H variant in men.

The role of the *BRCA2 *N372H variant on BC risk has been investigated by several studies in women from different populations with inconsistent findings that might reflect genetic differences across populations. In particular the HH genotype was associated with a 1.3- to 1.5-fold increased risk of BC in series from Northern Europe [8], Australia [9] and in a selected series of radiologic technologists from USA [10]. This association was not detected in series from Central Europe [13], Japan [12] and in an unselected series from USA [14].

Here, we performed a population-based case control study on male subjects all residing in the same area of Central Italy (Tuscany). Our analyses based on a recessive transmission model suggested an association between the *BRCA2 *N372H variant and increased MBC risk in men younger than 60 years. This effect on MBC risk was particularly evident when analyses were restricted to the sub-group of MBC cases with an interval between diagnosis and blood donation ≤ 4 years, in order to avoid a possible selection bias related to the enrolment of MBC cases with a longer survival in the attempt of expanding the series of patients affected with this rare disease. Overall, we had found a statistically significant interaction between N372H genotype and age: the effect on MBC risk of the HH homozygous genotype was significantly modified by age. In this respect, it is noteworthy that a recent pooled analysis with over 15,000 BC cases and 15,000 controls provided some evidence of an interaction of *BRCA2 *N372H with age, suggesting a possibly increased BC risk in women younger than 40 years of age [16].

It could be speculated that the effect of the *BRCA2 *N372H variant on BC risk could be modulated by the interactions with hormonal factors. In males, the hormonal background is not influenced by reproductive factors as in females and sex steroid levels decline with age [21]. Intriguingly, an association of the *BRCA2 *N372H variant with idiopathic male infertility, a condition possibly related to increased sensitivity to estrogens, has been recently suggested [22]. Here, we found that the *BRCA2 *N372H variant increased MBC risk in younger men that may show higher steroid hormones levels.

On the other hand, MBC risk could be influenced by interactions between genetic and environmental factors. Recently, we reported a possible modifying effect on MBC risk of an occupational exposure to chemicals, as polycyclic aromatic hydrocarbon, in subjects carrying *BRCA1/2 *germ-line mutations [23].

It has been also suggested that the *BRCA2 *N372H variant might affect fetal survival in a sex-dependent manner. In fact, an excess of NH heterozygotes in newborn females, compared to newborn males, has been observed and the *BRCA2 *N372H genotype distribution showed a significant deviation from the Hardy-Weinberg equilibrium in adult female controls with an excess of heterozygotes and a deficit of both homozygotes [8]. In our male population the overall distribution of the N372H genotypes fitted the Hardy-Weinberg equilibrium and the genotype frequencies were similar to those recently reported for a male population from UK [24] and for female populations from Central Europe and USA [13,14].

## Conclusion

Overall, our findings, although based on a relatively small series, suggest that the HH homozygous genotype might be positively associated with an increased risk of MBC in men younger than 60 years. This effect appears to be modified by age, with a statistically significant interaction. Due to the rarity of MBC, larger collaborative studies are needed to confirm our results in different male populations.

## Competing interests

The author(s) declare that they have no competing interests.

## Authors' contributions

DP participated in study design, coordination of field work, analysis and interpretation of data and critical review of the manuscript. MF performed SNP genotyping analyses and drafted the manuscript. GM and CS contributed to study design and statistical analyses. MC, MAC and IZ collected samples, clinical data and performed statistical analyses. RL, CDA, PR performed *BRCA1 *and *BRCA2 *mutational analysis. FS coordinated statistical analyses. LO participated in study design and supervision of experimental conduct and analysis, interpretation of results, drafting and revision of the manuscript, and approved the final version.

All authors read and approved the final manuscript.

## Pre-publication history

The pre-publication history for this paper can be accessed here:


